# Recombinant C1 inhibitor in the prevention of severe COVID-19: a randomized, open-label, multi-center phase IIa trial

**DOI:** 10.3389/fimmu.2023.1255292

**Published:** 2023-10-27

**Authors:** Pascal Urwyler, Marina Leimbacher, Panteleimon Charitos, Stephan Moser, Ingmar A. F. M. Heijnen, Marten Trendelenburg, Reto Thoma, Johannes Sumer, Adrián Camacho-Ortiz, Marcelo R. Bacci, Lars C. Huber, Melina Stüssi-Helbling, Werner C. Albrich, Parham Sendi, Michael Osthoff

**Affiliations:** ^1^ Division of Internal Medicine, University Hospital Basel, Basel, Switzerland; ^2^ Division of Medical Immunology, Laboratory Medicine, University Hospital Basel, Basel, Switzerland; ^3^ Department of Clinical Research, University of Basel, Basel, Switzerland; ^4^ Department of Biomedicine, University of Basel, Basel, Switzerland; ^5^ Division of Infectious Diseases and Hospital Epidemiology, Cantonal Hospital St. Gallen, St. Gallen, Switzerland; ^6^ Servicio de Infectologia, Hospital Universitario Dr. José Eleuterio González, Facultad de Medicina, Universidad Autónoma de Nuevo León, Monterrey, Mexico; ^7^ Department of General Practice, Centro Universitário em Saúde do ABC, Santo André, Brazil; ^8^ Clinic for Internal Medicine, City Hospital Triemli, Zurich, Switzerland; ^9^ Institute for Infectious Diseases, University of Bern, Bern, Switzerland

**Keywords:** COVID-19, randomized trial, C1 esterase inhibitor, complement system, kallikrein kinin system, contact activation system

## Abstract

**Background:**

Conestat alfa (ConA), a recombinant human C1 inhibitor, may prevent thromboinflammation.

**Methods:**

We conducted a randomized, open-label, multi-national clinical trial in which hospitalized adults at risk for progression to severe COVID-19 were assigned in a 2:1 ratio to receive either 3 days of ConA plus standard of care (SOC) or SOC alone. Primary and secondary endpoints were day 7 disease severity on the WHO Ordinal Scale, time to clinical improvement within 14 days, and safety, respectively.

**Results:**

The trial was prematurely terminated because of futility after randomization of 84 patients, 56 in the ConA and 28 in the control arm. At baseline, higher WHO Ordinal Scale scores were more frequently observed in the ConA than in the control arm. On day 7, no relevant differences in the primary outcome were noted between the two arms (*p* = 0.11). The median time to defervescence was 3 days, and the median time to clinical improvement was 7 days in both arms (*p* = 0.22 and 0.56, respectively). Activation of plasma cascades and endothelial cells over time was similar in both groups. The incidence of adverse events (AEs) was higher in the intervention arm (any AE, 30% with ConA vs. 19% with SOC alone; serious AE, 27% vs. 15%; death, 11% vs. 0%). None of these were judged as being related to the study drug.

**Conclusion:**

The study results do not support the use of ConA to prevent COVID-19 progression.

**Clinical trial registration:**

https://clinicaltrials.gov, identifier NCT04414631.

## 1 Introduction

Numerous efforts have been made to develop effective treatments for individuals who have been hospitalized with Coronavirus disease 2019 (COVID-19) (1). The pathogenesis of severe COVID-19 is related to SARS-CoV-2 infection and secondary to a dysregulated host immune response (2). In line with the excessive inflammatory response, coagulation activation plays a critical role (3, 4). Within the coagulation pathways, the importance of targeting the complement system (CS), the kinin–kallikrein system (KKS), and the contact activation system (CAS) to curb the progression of COVID-19 to severe disease has been supported by several observational studies, and the rationale to inhibit the CS with a therapeutic candidate has been outlined by us and others (5–9). Lower complement C1q values have been reported in patients with severe COVID-19, indicating relevant complement activation and consumption of this protein (10, 11). Within the CS, C1 inhibitor (C1INH) is a strong inhibitor of C1r/C1s and MASP-1/-2. In addition, C1INH also serves as an inhibitor of factor XII of the CAS as well as kallikrein of the KKS (12). Hence, C1INH may contribute to the deterrence of COVID-19 disease progression by prevention of microthrombi generation, inhibition of leucocyte activation, and interaction with endothelial cells and microorganisms (13, 14).

Conestat alfa (Ruconest^®^, Pharming, Leiden, The Netherlands, abbreviated as ConA in this manuscript) is a recombinant human C1INH and shares an identical protein structure with plasma-derived C1INH (pdC1INH). It has a different glycosylation pattern, which is responsible for a shorter half-life (3 h) than pdC1INH (30 h) (15, 16), and seems to target the activation of the lectin pathway more effectively (17). A pilot study using conestat alfa in five patients with severe COVID-19 showed promising results with immediate defervescence, and decrease of inflammatory markers and oxygen supplementation in four of five patients (18). To evaluate the efficacy and safety of ConA for SARS-CoV-2 infection, we conducted a randomized, open-label clinical trial in adult patients hospitalized with COVID-19 (PROTECT-COVID-19 trial).

## 2 Materials and methods

### 2.1 Study design and patients

This was a randomized, parallel-group, open-label, multi-center trial. The study protocol was published elsewhere (19). The study was performed in accordance with the Declaration of Helsinki and approved by the institutional review board and ethics committees for each participating institute. All participants gave written informed consent. An independent data-safety monitoring board (DSMB) committee periodically reviewed the study outcomes. The study was conducted at five sites in three countries (three sites in Switzerland and one site each in Brazil and Mexico) and registered at ClinicalTrials.gov (NCT04414631).

Male and non-pregnant female patients (18–85 years of age) were eligible and approached by study physicians of the participating centers if they had a diagnostic specimen that was positive for SARS-CoV-2 on reverse-transcriptase–polymerase-chain-reaction (RT-PCR), had pneumonia confirmed by chest imaging, and onset of symptoms ≤10 days or shortness of breath ≤5 days. The latter criterion was adapted/added with an amendment 4 months after approval of the first study protocol, because of the changing patient behaviors over time when seeking for medical help after a positive SARS-CoV-2 test. In addition, at least one of the following risk factors for disease progression was required for enrollment: arterial hypertension, age ≥ 50 years, obesity (BMI ≥ 30.0 kg/m^2^), cardiovascular disease, chronic pulmonary disease, chronic renal disease, C-reactive protein (CRP) of >35 mg/L, or oxygen saturation of ≤94%.

Exclusion criteria included refusal to participate, history or suspicion of allergy to rabbits or the study drug, pregnant or breast-feeding individuals, known severe liver disease, and renal disease requiring dialysis. Patients who received tocilizumab or another IL-6 or IL-6 receptor inhibitor prior to enrollment were not eligible for this study. Patients currently requiring intensive care (or expected to do so) within 24 h after initial presentation were not eligible for this study, because “prevention of disease progression” was investigated. Hospitalized patients requiring high-flow oxygen therapy on medical wards were eligible for inclusion.

### 2.2 Standard-of-care therapy, randomization, and study medication

*Standard of care (SOC) therapy*: All patients received SOC therapy. It was defined as any of the following treatment options, in addition to oxygen and fluid supply: dexamethasone, remdesivir, empiric antimicrobial treatment when bacterial co-infection was suspected, and anti-thrombotic treatment, according to in-house guidelines, aligned to published recommendations (20, 21). Other treatments such as tocilizumab, baricitinib, anti-SARS-CoV-2 monoclonal antibodies, and nirmatrelvir/ritonavir were not available or recommended in national guidelines during the conduct of the study.

#### 2.2.1 Randomization

Study participants were randomized in a 2:1 ratio using permuted-block allocation with varying block sizes. Randomization was stratified by the study site, generated by an independent statistician, and implemented via the electronic data capture system software SecuTrial^®^. There was no blinding of study medications.

#### 2.2.2 Intervention arm

Patients allocated to the intervention arm received a total of nine intravenous ConA doses over 72 h (8,400 U followed by 4,200 U every 8 h), in addition to SOC treatment.

#### 2.2.3 Control arm

Patients allocated to the control arm received SOC only.

### 2.3 Monitoring, outcomes, and follow-ups

Data on vital signs, disease severity according to WHO scale, clinical improvement, admission to ICU, and requirement for non-invasive or invasive ventilation were prospectively obtained. Virological clearance (time from positive to negative PCR test results in nasopharyngeal samples), hematological, liver, renal, and inflammatory parameters in laboratory examinations were monitored during the first 14 days of hospitalization or until discharge. C1INH antigen and C4 concentrations were determined on semi-automated platforms in the clinical laboratories in all except two study centers (not monitored in Monterrey, Mexico and Zurich, Switzerland). Additional EDTA- and citrate-plasma as well as serum samples were frozen at −80°C and subsequently analyzed for concentrations of C4d (Svar Life Sciences, Malmö, Sweden), sC5b-9 (BD Biosciences Pharmingen, San Diego, CA, USA), VCAM-1 and E-selectin (ProteinSimple, Bio-techne, Minneapolis, MN, USA), and kallikrein-like activity (Unitest, Haemochrome Diagnostica, Essen, Germany) according to the manufacturer’s instruction.

Final follow-up included a structured telephone interview or study visit (if still hospitalized) 28 days after enrollment to assess adverse events (AEs) and outcome. Afterwards, serious adverse events (SAEs) were followed until resolution, stabilization, or death, whichever occurred first.

The primary endpoint was disease severity on the WHO Ordinal Scale on day 7. For statistical analysis, the scores 6 and 7 were combined into one score. Secondary endpoint included time to clinical improvement within 14 days after enrollment. It was defined as improvement of two points on the WHO Ordinal Scale or live hospital discharge, whatever occurred first. Other secondary endpoints included the proportion of participants alive and not having required invasive or non-invasive ventilation at day 14 day, and the proportion of subjects with an acute lung injury. The latter was defined by the presence of PaO_2_/FiO_2_ ratio ≤300. Further endpoints included the length of hospital stay until day 28 in survivors, and all-cause mortality.

### 2.4 Safety evaluations

The overall incidence of AEs and SAEs were assessed during a 4-week follow-up and documented by the investigators according to seriousness, intensity, causal relationship with study treatment, action taken with study treatment (e.g., withdrawal), specific treatment for AE, and outcome.

AE and SAEs were grouped by the organ class system (22). There were no prespecified futility margins for the DSMB; however, the DSMB was entitled to recommend termination of the study.

### 2.5 Statistical plan and analysis

Full analysis set and intent-to-treat population (FAS/ITTP) was defined as all patients who were randomly allocated to a study arm. The primary efficacy analysis was based on the FAS/ITT population. The primary endpoint was analyzed by Wilcoxon test stratified by its baseline values with a two-sided α-level of 5%. The secondary endpoints were tested after a significant test of the primary endpoint (*a priori* ordered hypotheses); therefore, no alpha adjustment was necessary.

Quantitative secondary study parameters were described based on their mean, standard deviation (SD), median, interquartile range (IQR), minima, and maxima and portrayed by Kaplan–Meier plots and compared with the log rank test. Qualitative secondary study parameters were analyzed by means of absolute and relative frequencies: Chi-square tests were carried out in order to compare the active treatments to SOC. With a two-sided significance level of α = 0.05 and a power of 1 − β = 0.80, a sample size of 76 (2 × 38) was calculated for a fixed sample size design and an advantage of at least 1 point on the WHO Ordinal Scale (SD 1.5 points) (19). For a 2:1 randomization, a nonparametric analysis by the stratified Wilcoxon text, and an adaptive group sequential analysis, the overall sample size was estimated as 120 = 80 + 40. Two adaptive interim analyses after 40 and 80 patients were planned according to the Pocock adjusted levels α*p* = 0.0221. The analyses were carried out by the contract research organization CRM Biometrics GmbH (Bornheim, Germany).

The independent clinical trial statistician of CRM Biometrics GmbH, the first author (PU), and last author (MO) analyzed the data and had access to all data. All authors had access to primary clinical trial data only related to their institution. Study participants, treating physicians, nurses, investigators, and DSMB members were not blinded.

## 3 Results

### 3.1 Patient characteristics

Between August 2020 and March 2021, 621 patients were screened and 84 were enrolled (**Supplementary Figure S1**). Of these, 56 were randomized to the intervention and 28 to the control arm. One patient of the latter group withdrew consent after randomization (i.e., 27 for final analysis in the control arm). Screening failure reasons are cited in **Supplementary Table S1**. Fifty percent of the patients were recruited at the principal investigator’s institute (**Supplementary Table S2**). The baseline characteristics are presented in **Table 1**. Participants in the intervention arm presented with more severe disease at enrollment as assessed by the WHO scale score and the requirement of oxygen supplementation than the ones in the control arm, although the differences were not statistically significant. Corticosteroids such as dexamethasone were administered during the study in 42/56 (75%) and 19/27 (70%) of patients in the intervention and control arm, respectively.

**Table 1:** Baseline characteristics.

	Intervention arm (*n* = 56)	Control arm (*n* = 27)
Age (years), mean (SD)	61.1 (13.9)	64.4 (15.3)
Female gender, n (%)	17 (30)	10 (36)
BMI (kg/m^2^), mean (SD)	29.1 (6.1)	26.4 (4.5)
Shortness of breath, n (%)	42 (75)	15 (54)
Fever, n (%)	36 (64)	13 (46)
Cough, n (%)	43 (77)	18 (64)
Arterial hypertension, n (%)	27 (49)	15 (54)
COPD/chronic lung disease, n (%)	16 (29)	6 (22)
Cardiovascular disease, n (%)	12 (21)	6 (22)
Diabetes mellitus, n (%)	15 (27)	8 (29)
Heart failure, n (%)	5 (9)	4 (14)
Smoking (past and present), n (%)	27 (48)	8 (30)
Anticoagulation, n (%)	10 (18)	4 (14)
ACE/ARB, n (%)	23 (41)	13 (48)
Oxygen supplementation (L), mean (SD)	4.6 (8.4)	2.1 (2.1)
WHO Ordinal Scale score at enrolment		
Score 3	12 (21)	10 (37)
Score 4	41 (73)	16 (59)
Score 5	3 (5)	2 (4)

ACE, angiotension-converting enzyme; ARB, angiotension receptor blocker; BMI, body mass index; COPD, chronic obstructive pulmonary disease; SD, standard deviation.

### 3.2 Endpoints

**Figure 1:**
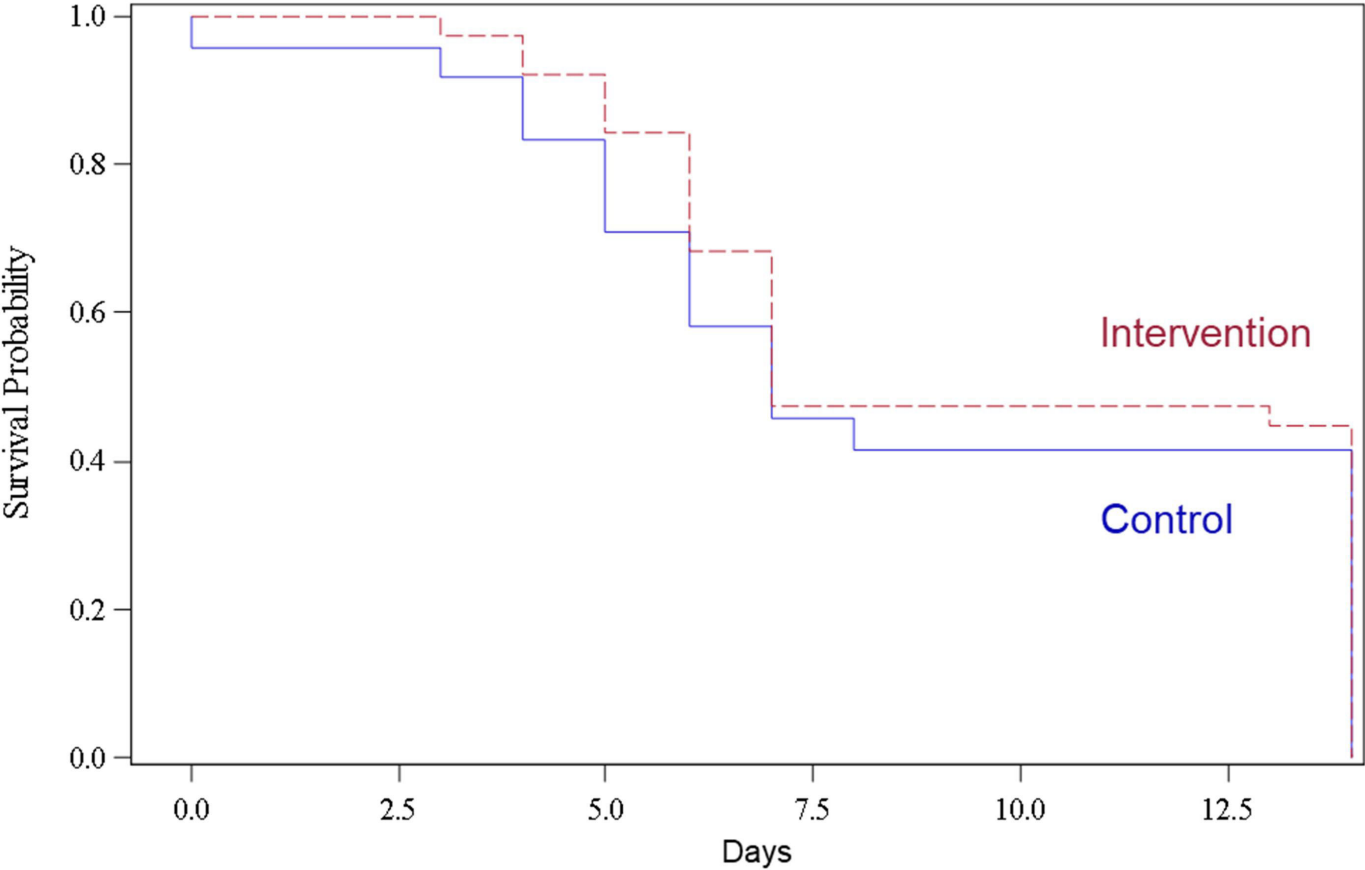
Kaplan–Meier curve for the time to clinical improvement within 14 days according to the treatment group.

Disease severity on day 7, the primary endpoint, was not different in the two groups (*p* = 0.11) (**Table 2**), which was also the case after stratification by the baseline disease severity score (**Table 3**), and when the change of the WHO Ordinal Scale from baseline to day 7 was analyzed (*p* = 0.96, data not shown). Consistent with the observations at baseline, severe disease presentations were more frequently observed at day 7 in the intervention than in the control arm (19.7% vs. 7.4%, *p* = 0.21). The median time to clinical improvement was 7 days in both arms (*p* = 0.57) (**Figure 1**). Patients in the intervention arm more frequently required mechanical ventilation within 14 days (*n* = 8, 14.3%) and developed more often acute lung injury (*n* = 3, 5.4%) than the ones in the control arm (*n* = 1, 3.7% and *n* = 0, respectively, *p* = 0.26 for the comparison of mechanical ventilation). The median time to defervescence was 3.0 days in both arms (*p* = 0.22). The analysis of disease severity on day 14 mirrored the results on day 7 with no differences in the two groups (*p* = 0.40). Admission to the ICU until day 14 was required in 10 (17.9%) and 2 (7.4%) patients in the intervention and control arm, respectively (*p* = 0.32). The proportions of patients discharged at day 14 were 38 (67.9%) and 23 (85.2%) in the intervention and control arm, respectively. The length of hospital stay in survivors was longer in the intervention arm (mean 11.4 days, SD 8.0) in comparison to the one in the control arm (mean 7.6 days, SD 5.2, *p* = 0.04). During the 28-day follow-up, death was only observed in the intervention arm (*n* = 6; three, two, and one case in Switzerland, Brazil, and Mexico, respectively). Two additional deaths in the intervention arm and one in the control arm were noted during the subsequent follow-up period of SAEs.

**Table 2:** Disease severity at day 7.

	WHO Ordinal Scale (score points)	Intervention arm (*n* = 56)	Control arm (*n* = 27)
Outpatient, n (%)	1	8 (14.3)	7 (25.9)
	2	12 (21.4)	5 (18.5)
Hospitalized—mild disease, n (%)	3	13 (23.2)	5 (18.5)
	4	12 (21.4)	8 (29.6)
Hospitalized—severe disease, n (%)	5	3 (5.4)	1 (3.7)
	6	6 (10.7)	1 (3.7)
Death, n (%)	7	2 (3.6)	–
*p*-value (Wilcoxon test stratified by baseline)		0.11	

**Table 3:** Disease severity on day 7 stratified by baseline scores.

WHO Ordinal Scale			Intervention arm (*n* = 56)	Control arm (*n* = 27)	
Baseline (score points)	Day 7 (score points)				
3	*n*		12	10
	1	*n* (%)	3 (25.0)	6 (60.0)
	2		6 (50.0)	1 (10.0)
	3		2 (16.7)	2 (20.0)
	4		4 (8.3)	1 (10.0)
	*p*-value		0.38	
≥4	n		44	17
	1	*n* (%)	5 (11.4)	1 (5.9)
	2		6 (13.6)	4 (23.5)
	3		11 (25.0)	3 (17.7)	
	4		11 (25.0)	7 (41.2)	
	5		3 (6.8)	1 (5.9)	
	6		6 (13.6)	1 (5.9)	
	7		2 (4.6)	–	
	*p*-value		0.70	

### 3.3 Laboratory analyses

**Figure 2:**
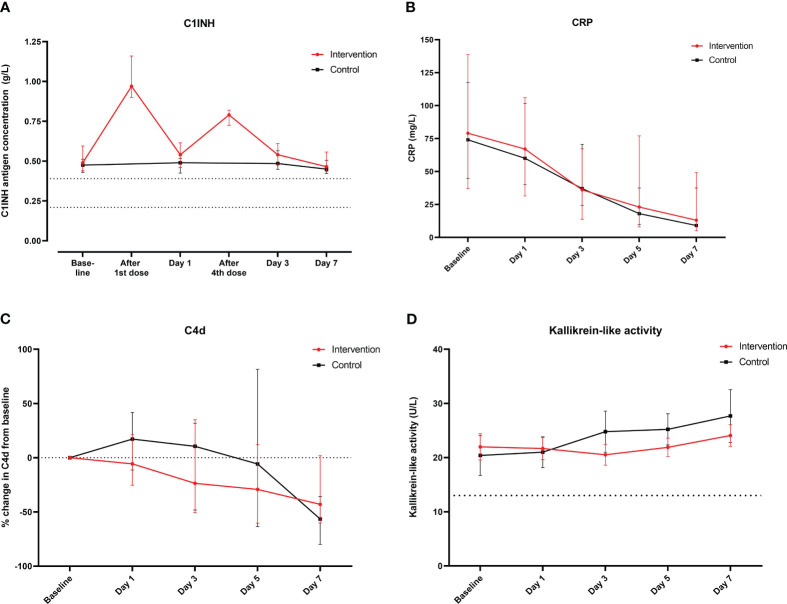
Course of main laboratory parameters from baseline until day 7 according to the treatment group. Course of **(A)** C1INH concentrations (g/L, medians and interquartile ranges; dashed lines indicate the normal range in healthy adults), **(B)** CRP (mg/L, medians and interquartile ranges), and **(C)** relative change in C4d (% change from baseline, medians and interquartile ranges; dashed line indicates baseline) and **(D)** course of kallikrein-like activity (U/L, medians and interquartile ranges; dashed line indicates activity of control plasma).

Analysis of C1INH concentrations revealed elevated values in both groups at baseline (**Figure 2A**). They nearly doubled after the first ConA administration (8,400 U) in the intervention arm, but returned close to baseline before the 4th administration (4,200 U). Subsequent dose adminstrations increased the C1INH concentrations each by approximately 50%. The mean time until SARS-CoV-2 clearance was 6.3 (SD 4.1) and 6.4 (SD 2.4) days in the intervention and control arm, respectively (*p* = 0.74). Similarly, the course of CRP (**Figure 2B**), ferritin, D-dimer, creatinine (**Supplementary Figure S2**), and lymphocyte values (data not shown) was similar in both groups. There was no evidence of relevant complement C4 consumption (**Supplementary Figure S3**) and no difference in the activation of the CS (as measured by C4d and sC5b-9, respectively), endothelial cells (as measured by VCAM-1 and E-selectin), or the KSS (as measured by kallikrein-like activity) in both groups (**Figure 2**; **Supplementary Figure S3**).

### 3.4 Safety

#### 3.4.1 Adverse events

Twenty-two subjects had at least one AE: 17 (30%) in the intervention arm and 5 (19%) in the control arm. Overall, 35 AEs were reported: 30 (85.7%) in the interventon arm and 5 (14.3%) in the control arm. Ten of these AEs were attributed to investigations [i.e., laboratory parameters: eight (80.0%) in the intervention arm and two (20.0%) in the control arm]. Details are listed in **Supplementary Tables S3**and **S4**.

#### 3.4.2 Serious adverse events

These occurred in 15 (27%) patients in the intervention arm and 4 (15%) in the control arm. The most common system organ classes of the SAEs were “respiratory, thoracic and mediastinal disorders” [10 (17.9%) and 2 (7.4%)], “infections and infestations” [6 (10.7%) and 1 (3.7%)], and “cardiac disorders” [3 (5.4%) and 0]. Details are listed in **Supplementary Table S5**.

None of the AEs or SAEs were judged as being related to the study drug by the investigators.

### 3.5 Premature trial determination

While SAEs and deaths were judged as not related to the trial medication, their increased occurrence in the intervention arm led the DSMB to recommend an interruption of the trial after the second interim analysis. In addition, the DSMB recommended to await the outcome data of this analysis, including analyses of the activity of the plasma cascades prior to further decision-making on cessation or continuation of the trial. Subsequently, the trial was terminated prematurely in September 2021 for the following reasons. First, the intervention regimen was not associated with a significant inhibition of the CS and activation of other plasma cascades or endothelial cells. Second, considering the results in the primary outcome not favoring the intervention and the observed imbalances in the baseline characteristics, a larger sample size would have been required to show a difference if there was a true difference. Third, during the second half of the year 2021, additional treatment options such as anti-SARS-CoV-2 monoclonal antibodies and tocilizumab were introduced and recommended, making the study groups even more heterogeneous.

## 4 Discussion

This randomized trial found that C1INH treatment added to SOC was not associated with clinical improvement or faster recovery from COVID-19 in comparison with SOC alone in hospitalized, non-critically ill patients. The overall mortality in this trial (7%) was substantially lower than the one previously reported in the dexamethasone trial (23%), published by the RECOVERY collaborative group (23). The current trial was terminated prematurely because of a lack of efficacy. The power of the study is low because the sample size needed for adjustment after the interim analyses could not be realized. Although there were more AEs and SAEs in the intervention arm than in the control arm, the trial was not terminated because of safety concerns.

The conduction of this trial was justified by scientific rationales and a previous pilot study (18). The former included the idea that C1INH may dampen uncontrolled complement activation and collateral lung damage, may reduce capillary leakage and subsequent pulmonary edema and the generation of microthrombi by inhibition of the KKS and the CAS, and may preserve the regulatory role of endothelial cells. Despite these arguments, we did not observe a clinical benefit in preventing COVID-19 progression when using ConA plus SOC. The following reasons may explain the study outocomes observed.

First, the administration of ConA may have been too late in the course of COVID-19. Because of the short half-life of ConA, repeated administration of ConA over 3 days was chosen. The dosage chosen was supposed to increase the C1INH concentrations by at least 50%. Pharmacokinetic analyses revealed that C1INH concentrations were already markedly elevated at baseline in both arms, which is in agreement with our previous data (24). It is also a potential hint that activation of plasma cascades is already significant and potentially beyond the “point of no return” for inhibition by C1INH, when patients with COVID-19 seek for medical help because of their symptoms. C1INH targets plasma cascades at an early activation level.

Second, the dosing regimen may have been unable to inhibit the CS in COVID-19 sufficiently and for a prolonged period of time. C1INH concentrations doubled initially after the first administration of ConA but returned to almost baseline before the 4th administration. The exact concentration required to inhibit the complement and other cascades and the optimal target are unknown both in the specific context of COVID-19 and in hyperinflammation secondary to infection in general. Of note, COVID-19 is characterized by a local activation of plasma cascades that progresses to a systemic activation early in the disease (25). As such, our data demonstrate that even a twofold increase in C1INH concentration is not sufficient to significantly inhibit all potential downstream effects of the CS, KKS, CAS, and endothelial cells (26, 27).

Third, in COVID-19, the potential inhibitory role of C1INH may have been overestimated when considering activation through the classical and alternative pathway (11, 24, 28, 29). The lectin pathway of complement has been initially implicated as a major driver of complement activation, and binding of MBL or MASP-2 to SARS-CoV-2 was demonstrated with subsequent complement activation (30–32). Subsequently, it became clear that the S protein of SARS-CoV-2 directly activates the alternative pathway (33), which is not efficiently inhibited by C1INH, and circulating immune complexes may activate the classical pathway (28), in line with results from autopsy studies (34).

Fourth, cross-activation of the CS downstream of the lectin and classical pathway proteases may play a considerable role in COVID-19 disease progression. For example, factor XIa inhibited the regulatory complement factor H of the alternative pathway enhancing alternative pathway activity (35). Activated platelets may trigger alternative pathway activation (36–38), and the proteases of the coagulation and fibrinolysis cascades (both activated during COVID-19 but not or only poorly influenced by C1INH) are capable of cross-activating the CS at the level of C3 or C5 (39, 40). In line, the course of serum markers of inflammation and activation of the coagulation system was similar in both arms. Last, it is unclear if different SARS-CoV-2 variants circulating during the study period may have differential effects on the activation of plasma cascade and hence the inhibitory effect of C1INH.

The CS has been a target of a number of randomized controlled trials in COVID-19. However, only retrospective data from patients requiring continuous positive airway pressure support (41) and a single phase 3 trial reported a survival benefit when administering an anti-C5a antibody, albeit in mechanically ventilated COVID-19 patients for the latter (42), whereas another large phase 3 trial in the same population using a C5 inhibitor was futile (43). Clinical trials targeting the CAS and KKS are scarce, and consist of small sample sizes (44, 45). In the only published randomized controlled trial of C1INH in COVID-19, C1INH did not influence clinical improvement and length of stay compared to the SOC arm, in line with our study results (46). The present trial, despite reporting negative findings, is important to direct future clinical interventions targeting the CS, KKS, and CAS in other respiratory viral diseases.

This trial was designed early during the pandemic. As such, it was decided that instead of defining futility margins for a rather small-scale trial including a diverse patient population, a regular review of the DSMB might be more appropriate, as the included patients would potentially receive various new treatments with poorly known safety profiles as time evolved. Instead, two adaptive interim analyses after 40 and 80 patients were carried out. Although more SAEs occurred in the intervention arm, the majority of them were noted after the intervention period. In agreement with external evaluations, we attributed most SAEs to COVID-19 or a complication of it (e.g., pneumonia or sepsis). This interpretation is also supported by the observation of more severe COVID-19 at baseline and throughout the study period in the intervention arm. Of note and in contrast to C5 inhibitory strategies, C1INH treatment has not been associated with an increased risk for bacterial or viral infections or any effect on viral replication (47). Despite being implemented in the electronic case report form and using variable block sizes, which impairs correct forecasting of the allocation, randomization was not associated with a balanced distribution of severity. Reasons include the 2:1 randomization ratio associated with a rather small number of individuals included in the control arm. Moreover, stratification by center may have contributed to the imbalances observed given that some centers only enrolled a limited number of patients. Indeed, we observed a much larger imbalance after the first interim analysis (40 patients included), which was reduced after including more patients. The DSMB reviewed all safety data and deaths in detail during three meetings. For several reasons including the imbalances in disease severity at baseline favoring the control group and the overall lower-than-expected mortality in the intervention arm in the Swiss centers with the majority of enrolled patients, termination of the study for safety was not recommended.

Our study has limitations. These include the premature closure of the trial, the lack of prespecified futility margins, the small number of patients in the control arm (due to the 2:1 randomization), and the imbalances in baseline characteristics and disease severity observed between the groups at baseline (potentially due to the 2:1 randomization and stratification in the setting of a limited number of enrolled patients). Almost 50% of patients were recruited in one center, reflecting an uneven distribution between the institutions. It was an open-label trial, and as such, it may have been prone to bias. The SOC treatment allowed the use of remdesivir and dexamethasone, which may have diminished the possible effect of ConA. Last but not least, follow-up phone calls might potentially have been less accurate than in-person follow-up visits when assessing for AEs. Despite these limitations, this is the largest study to investigate a treatment strategy that interferes with the CS, KKS, and CAS in hospitalized COVID-19 patients. In addition, we provide patient-matched laboratory data regarding the inability of ConA to limit the activation of these plasma cascades in COVID-19.

In conclusion, ConA did not significantly accelerate clinical improvement or reduce mortality in hospitalized patients with non-critically ill COVID-19 infection. The chosen dose and regimen of ConA used in this study are insufficient to limit complement activation in COVID-19. It remains unclear whether or not higher dosages, a longer treatment regimen, or an earlier intervention time point with ConA in COVID-19 could have prevented disease progression.

## Data availability statement

The raw data supporting the conclusions of this article will be made available by the authors, on reasonable request.

## Ethics statement

The studies involving humans were approved by the ethics committees «Ethikkommission Nordwest- und Zentralschweiz», «Ethikkommission Ostschweiz», and “Kantonale Ethikkommission Zürich» in Switzerland, “Comissâo Nacional de Ética Em Pesquisa” (CONEP) in Brazil and “Comisión Federal Para La Protección Contra Riesgos Sanitarios” (COFEPRIS) in Mexico. The studies were conducted in accordance with the local legislation and institutional requirements. The participants provided their written informed consent to participate in this study.

## Author contributions

PU: Conceptualization, Data curation, Formal Analysis, Investigation, Methodology, Project administration, Supervision, Visualization, Writing – original draft, Writing – review & editing. ML: Investigation, Project administration, Writing – review & editing. PC: Investigation, Project administration, Writing – review & editing. SM: Investigation, Project administration, Writing – review & editing. IH: Conceptualization, Investigation, Methodology, Writing – review & editing. MT: Investigation, Project administration, Supervision, Writing – review & editing. RT: Investigation, Project administration, Writing – review & editing. JS: Investigation, Project administration, Writing – review & editing. AC: Investigation, Project administration, Supervision, Writing – review & editing. MB: Investigation, Project administration, Supervision, Writing – review & editing. LH: Investigation, Project administration, Supervision, Writing – review & editing. MS: Investigation, Project administration, Writing – review & editing. WA: Investigation, Project administration, Supervision, Writing – review & editing. PS: Conceptualization, Formal Analysis, Investigation, Methodology, Project administration, Supervision, Validation, Writing – original draft, Writing – review & editing. MO: Conceptualization, Data curation, Formal Analysis, Funding acquisition, Investigation, Methodology, Project administration, Resources, Supervision, Validation, Visualization, Writing – original draft, Writing – review & editing.

## References

[1] Narota A Puri G Singh VP Kumar A Naura AS. COVID-19 and ARDS: Update on preventive and therapeutic venues. Curr Mol Med (2022) 22(4):312–24. doi: 10.2174/1566524021666210408103921 33829971

[2] Boechat JL Chora I Morais A Delgado L. The immune response to SARS-CoV-2 and COVID-19 immunopathology - Current perspectives. Pulmonology. (2021) 27(5):423–37. doi: 10.1016/j.pulmoe.2021.03.008 PMC8040543 33867315

[3] Opal SM. Phylogenetic and functional relationships between coagulation and the innate immune response. Crit Care Med (2000) 28(9 Suppl):S77–80. doi: 10.1097/00003246-200009001-00017 11007204

[4] Ackermann M Verleden SE Kuehnel M Haverich A Welte T Laenger F. Pulmonary vascular endothelialitis, thrombosis, and angiogenesis in covid-19. N Engl J Med (2020) 383(2):120–8. doi: 10.1056/NEJMoa2015432 PMC7412750 32437596

[5] Bekassy Z Lopatko Fagerstrom I Bader M Karpman D. Crosstalk between the renin-angiotensin, complement and kallikrein-kinin systems in inflammation. Nat Rev Immunol (2021). doi: 10.1038/s41577-021-00634-8 PMC8579187 34759348

[6] Holter JC Pischke SE de Boer E Lind A Jenum S Holten AR. Systemic complement activation is associated with respiratory failure in COVID-19 hospitalized patients. Proc Natl Acad Sci USA (2020) 117(40):25018–25. doi: 10.1073/pnas.2010540117 PMC7547220 32943538

[7] de Nooijer AH Grondman I Janssen NAF Netea MG Willems L van de Veerdonk FL. Complement activation in the disease course of COVID-19 and its effects on clinical outcomes. J Infect Dis (2020). doi: 10.1093/infdis/jiaa646 PMC7797765 33038254

[8] Carvelli J Demaria O Vely F Batista L Benmansour NC Fares J. Association of COVID-19 inflammation with activation of the C5a-C5aR1 axis. Nature (2020). doi: 10.1136/jitc-2020-SITC2020.0483 PMC7116884 32726800

[9] D'Alessandro A Thomas T Dzieciatkowska M Hill RC Francis RO Hudson KE. Serum proteomics in COVID-19 patients: altered coagulation and complement status as a function of IL-6 level. J Proteome Res (2020) 19(11):4417–27. doi: 10.1021/acs.jproteome.0c00365 PMC7640953 32786691

[10] Wu Y Huang X Sun J Xie T Lei Y Muhammad J. Clinical characteristics and immune injury mechanisms in 71 patients with COVID-19. mSphere (2020) 5(4). doi: 10.1128/mSphere.00362-20 PMC7364211 32669467

[11] Sinkovits G Mező B Réti M Müller V Iványi Z Gál J. Complement overactivation and consumption predicts in-hospital mortality in SARS-coV-2 infection. Front Immunol (2021) 12:663187. doi: 10.3389/fimmu.2021.663187 33841446 PMC8027327

[12] Cicardi M Zingale L Zanichelli A Pappalardo E Cicardi B. C1 inhibitor: molecular and clinical aspects. Springer Semin Immunopathol (2005) 27(3):286–98. doi: 10.1007/s00281-005-0001-4 16267649

[13] Cai S Dole VS Bergmeier W Scafidi J Feng H Wagner DD. A direct role for C1 inhibitor in regulation of leukocyte adhesion. J Immunol (2005) 174(10):6462–6. doi: 10.4049/jimmunol.174.10.6462 15879149

[14] Renné T Pozgajová M Grüner S Schuh K Pauer HU Burfeind P. Defective thrombus formation in mice lacking coagulation factor XII. J Exp Med (2005) 202(2):271–81. doi: 10.1084/jem.20050664 PMC2213000 16009717

[15] Davis B Bernstein JA. Conestat alfa for the treatment of angioedema attacks. Ther Clin Risk Manage (2011) 7:265–73. doi: 10.2147/TCRM.S15544 PMC3132097 21753889

[16] van Veen HA Koiter J Vogelezang CJ van Wessel N van Dam T Velterop I. Characterization of recombinant human C1 inhibitor secreted in milk of transgenic rabbits. J Biotechnol (2012) 162(2-3):319–26. doi: 10.1016/j.jbiotec.2012.09.005 22995741

[17] Gesuete R Storini C Fantin A Stravalaci M Zanier ER Orsini F. Recombinant C1 inhibitor in brain ischemic injury. Ann neurology (2009) 66(3):332–42. doi: 10.1002/ana.21740 19798727

[18] Urwyler P Moser S Charitos P Heijnen I Rudin M Sommer G. Treatment of COVID-19 with conestat alfa, a regulator of the complement, contact activation and kallikrein-kinin system. Front Immunol (2020) 11:2072. doi: 10.3389/fimmu.2020.02072 32922409 PMC7456998

[19] Urwyler P Charitos P Moser S Heijnen I Trendelenburg M Thoma R. Recombinant human C1 esterase inhibitor (conestat alfa) in the prevention of severe SARS-CoV-2 infection in hospitalized patients with COVID-19: A structured summary of a study protocol for a randomized, parallel-group, open-label, multi-center pilot trial (PROTECT-COVID-19). Trials. (2021) 22(1):1. doi: 10.1186/s13063-020-04976-x 33397449 PMC7780206

[20] IDSA. IDSA Guidelines on the Treatment and Management of Patients with COVID-19 idsociety.org. Arlington, VA 22203, USA: IDSA (2022). Available at: https://www.idsociety.org/practice-guideline/covid-19-guideline-treatment-and-management/#SupplementaryInformation.

[21] Cuker A Tseng EK Schünemann HJ Angchaisuksiri P Blair C Dane K. American Society of Hematology living guidelines on the use of anticoagulation for thromboprophylaxis for patients with COVID-19: March 2022 update on the use of anticoagulation in critically ill patients. Blood Adv (2022) 6(17):4975–82. doi: 10.1182/bloodadvances.2022007940 PMC9236618 35748885

[22] Carragher R Robertson C. Assessing safety at the end of clinical trials using system organ classes: A case and comparative study. Pharm Stat (2021) 20(6):1278–87. doi: 10.1002/pst.2148 34169636

[23] Horby P Lim WS Emberson JR Mafham M Bell JL Linsell L. Dexamethasone in hospitalized patients with covid-19. N Engl J Med (2021) 384(8):693–704. doi: 10.1056/NEJMoa2021436 32678530 PMC7383595

[24] Charitos P Heijnen I Egli A Bassetti S Trendelenburg M Osthoff M. Functional activity of the complement system in hospitalized COVID-19 patients: A prospective cohort study. Front Immunol (2021) 12:765330. doi: 10.3389/fimmu.2021.765330 34777382 PMC8581394

[25] Busch MH Timmermans S Nagy M Visser M Huckriede J Aendekerk JP. Neutrophils and contact activation of coagulation as potential drivers of COVID-19. Circulation. (2020) 142(18):1787–90. doi: 10.1161/CIRCULATIONAHA.120.050656 PMC7594534 32946302

[26] Peoples N Strang C. Complement activation in the central nervous system: A biophysical model for immune dysregulation in the disease state. Front Mol Neurosci (2021) 14:620090. doi: 10.3389/fnmol.2021.620090 33746710 PMC7969890

[27] Ravindran S Grys TE Welch RA Schapira M Patston PA. Inhibition of plasma kallikrein by C1-inhibitor: role of endothelial cells and the amino-terminal domain of C1-inhibitor. Thromb Haemost (2004) 92(6):1277–83. doi: 10.1160/TH04-01-0008 15583734

[28] Castanha PMS Tuttle DJ Kitsios GD Jacobs JL Braga-Neto U Duespohl M. IgG response to SARS-CoV-2 and seasonal coronaviruses contributes to complement overactivation in severe COVID-19 patients. J Infect Dis (2022). doi: 10.1093/infdis/jiac091

[29] Lipcsey M Persson B Eriksson O Blom AM Fromell K Hultstrom M. The outcome of critically ill COVID-19 patients is linked to thromboinflammation dominated by the kallikrein/kinin system. Front Immunol (2021) 12:627579. doi: 10.3389/fimmu.2021.627579 33692801 PMC7937878

[30] Gao T Hu M Zhang X. Highly pathogenic coronavirus N protein aggravates lung injury by MASP-2-mediated complement over-activation. Signal Transduct Target Ther (2020). 7:318 doi: 10.1101/2020.03.29.20041962 PMC9470675 36100602

[31] Magro C Mulvey JJ Berlin D Nuovo G Salvatore S Harp J. Complement associated microvascular injury and thrombosis in the pathogenesis of severe COVID-19 infection: A report of five cases. Transl Res (2020) 220:1–13. doi: 10.1016/j.trsl.2020.04.007 32299776 PMC7158248

[32] Stravalaci M Pagani I Paraboschi EM Pedotti M Doni A Scavello F. Recognition and inhibition of SARS-CoV-2 by humoral innate immunity pattern recognition molecules. Nat Immunol (2022) 23(2):275–86. doi: 10.1038/s41590-021-01114-w 35102342

[33] Yu J Yuan X Chen H Chaturvedi S Braunstein EM Brodsky RA. Direct activation of the alternative complement pathway by SARS-CoV-2 spike proteins is blocked by factor D inhibition. Blood. (2020) 136(18):2080–9. doi: 10.1182/blood.2020008248 PMC7596849 32877502

[34] Macor P Durigutto P Mangogna A Bussani R De Maso L D'Errico S. Multiple-organ complement deposition on vascular endothelium in COVID-19 patients. Biomedicines (2021) 9(8). doi: 10.3390/biomedicines9081003 PMC8394811 34440207

[35] Puy C Pang J Reitsma SE Lorentz CU Tucker EI Gailani D. Cross-talk between the complement pathway and the contact activation system of coagulation: activated factor XI neutralizes complement factor H. J Immunol (2021) 206(8):1784–92. doi: 10.4049/jimmunol.2000398 PMC8030746 33811105

[36] Del Conde I Cruz MA Zhang H Lopez JA Afshar-Kharghan V. Platelet activation leads to activation and propagation of the complement system. J Exp Med (2005) 201(6):871–9. doi: 10.1084/jem.20041497 PMC2213112 15781579

[37] Peerschke EI Valentino A So RJ Shulman S Ravinder. Thromboinflammation supports complement activation in cancer patients with COVID-19. Front Immunol (2021) 12:716361. doi: 10.3389/fimmu.2021.716361 34491250 PMC8416543

[38] Peerschke EI Yin W Ghebrehiwet B. Complement activation on platelets: implications for vascular inflammation and thrombosis. Mol Immunol (2010) 47(13):2170–5. doi: 10.1016/j.molimm.2010.05.009 PMC2904326 20621693

[39] Amara U Flierl MA Rittirsch D Klos A Chen H Acker B. Molecular intercommunication between the complement and coagulation systems. J Immunol (2010) 185(9):5628–36. doi: 10.4049/jimmunol.0903678 PMC3123139 20870944

[40] Kanse SM Gallenmueller A Zeerleder S Stephan F Rannou O Denk S. Factor VII-activating protease is activated in multiple trauma patients and generates anaphylatoxin C5a. J Immunol (2012) 188(6):2858–65. doi: 10.4049/jimmunol.1103029 22308306

[41] Ruggenenti P Di Marco F Cortinovis M Lorini L Sala S Novelli L. Eculizumab in patients with severe coronavirus disease 2019 (COVID-19) requiring continuous positive airway pressure ventilator support: Retrospective cohort study. PloS One (2021) 16(12):e0261113. doi: 10.1371/journal.pone.0261113 34928990 PMC8687582

[42] Vlaar APJ Witzenrath M van Paassen P Heunks LMA Mourvillier B de Bruin S. Anti-C5a antibody (vilobelimab) therapy for critically ill, invasively mechanically ventilated patients with COVID-19 (PANAMO): a multicentre, double-blind, randomised, placebo-controlled, phase 3 trial. Lancet Respir Med (2022) 10(12):1137–46. doi: 10.1016/S2213-2600(22)00297-1 PMC9451499 36087611

[43] Annane D Pittock SJ Kulkarni HS Pickering BW Khoshnevis MR Siegel JL. Intravenous ravulizumab in mechanically ventilated patients hospitalised with severe COVID-19: a phase 3, multicentre, open-label, randomised controlled trial. Lancet Respir Med (2023). doi: 10.1016/S2213-2600(23)00082-6 PMC10027334 36958364

[44] Malchair P Giol J Garcia V Rodriguez O Ruibal JC Zarauza A. Three-day icatibant on top of standard care in patients with COVID-19 pneumonia (ICAT.COVID): a randomized, open-label, phase 2, proof-of-concept trial. Clin Infect Dis (2023). doi: 10.1093/cid/ciac984 PMC10209439 36610464

[45] Mansour E Palma AC Ulaf RG Ribeiro LC Bernardes AF Nunes TA. Safety and outcomes associated with the pharmacological inhibition of the kinin-kallikrein system in severe COVID-19. Viruses (2021) 13(2). doi: 10.3390/v13020309 PMC7920028 33669276

[46] Pagano L Salmanton-Garcia J Marchesi F Busca A Corradini P Hoenigl M. COVID-19 infection in adult patients with hematological Malignancies: a European Hematology Association Survey (EPICOVIDEHA). J Hematol Oncol (2021) 14(1):168. doi: 10.1186/s13045-021-01177-0 34649563 PMC8515781

[47] Bork K Steffensen I Machnig T. Treatment with C1-esterase inhibitor concentrate in type I or II hereditary angioedema: a systematic literature review. Allergy Asthma Proc (2013) 34(4):312–27. doi: 10.2500/aap.2013.34.3677 23710659

