# Supplementary material for: Recombinant C1 inhibitor in the prevention of severe COVID-19: a randomized, open-label, multi-center phase IIa trial
Source: Front Immunol. 2023 Oct 27;14:1255292. doi: 10.3389/fimmu.2023.1255292 (PMC10641758; doi:10.3389/fimmu.2023.1255292)
Supplement: Supplementary file 1 [file DataSheet_1.docx]

Supplementary Material

Table of Contents

| **Supplementary Figure S1.** Flow diagram of the study | Page 2 |
| --- | --- |
| **Supplementary Table ST1.** Screening failure reasons. Multiple citations possible | Page 3 |
| **Supplementary Table ST2.** Patients’ distribution according to the enrollment center | Page 4 |
| **Supplementary Figure S2.** Course of additional laboratory parameters from baseline until day 7 according to the treatment group | Page 5 |
| **Supplementary Figure S3.** Course of complement and endothelial activation parameters from baseline until day 7 according to the treatment group | Page 6 |
| **Supplementary Table ST3.** Adverse events classified by System Organ Classification | Page 7 |
| **Supplementary Table ST4.** Adverse events by preferred term of the investigators | Page 8 |
| **Supplementary Table ST5.** Serious adverse events classified by System Organ Classification and preferred term by the investigator | Page 9 |

Supplementary Figure S1. Flow diagram of the study.

Assessed for eligibility (n=621)

## Enrollment

Excluded (n=537)

- For detailed listing of exclusion and inclusion see Supplementary Table ST1

Randomized (n=84)

**Intervention arm (n=56)**

- Received allocated intervention (n=56)

## Allocation

**Control arm (n=28)**

- Consent withdrawn after enrollment (n=1)
- Deaths (n=6)
- Drop-outs (n=2)
- Lost to follow-up (n=0)
- Deaths (n=0)
- Drop-outs (n=0)
- Lost to follow-up (n=0)

## Follow-Up

## Analysis

## (Intention to treat)

Analyzed (n=27)

Analyzed (n=56)

# Supplementary Table ST1. Screening failure reasons. Multiple citations possible.

| Category | Criterion | Number of individuals |
| --- | --- | --- |
| Inclusion criterion |  |  |
|  | Age 18-85 years | 74 |
|  | Positive SARS-CoV-2 PCR | 26 |
|  | Evidence of pulmonary involvement on CT scan or X-ray | 151 |
|  | Symptom onset within previous 10 days | 206 |
|  | Expected to remain inpatient for next 3 calendar days | 107 |
|  | At least one additional risk factor | 20 |
| Exclusion criterion |  |  |
|  | Contraindication to C1 inhibitor (e.g. known hypersensitivity or allergy to class of drugs) | 6 |
|  | History or suspicion of allergy to rabbits | 8 |
|  | Treatment with tocilizumab or another IL-6 inhibitor | 7 |
|  | Active or planned treatment with any complement inhibitor | 6 |
|  | Liver cirrhosis (any Child-Pugh score) | 15 |
|  | Incapacity or inability to provide informed consent | 147 |
|  | Currently admitted to an ICU or expected admission within the next 24 hours | 26 |
|  | Currently receiving invasive or non-invasive ventilation | 10 |
|  | In the opinion of the treating team, death is deemed to be imminent and inevitable within the next 24 hours | 8 |
|  | Participation in another study with investigational drug within the 30 days preceding and during the study | 11 |
|  | Previous enrollment into the current study | 5 |
|  | Enrollment of the investigator, his/her family members, employees and other dependent persons | 7 |
|  | Women who are pregnant or breast feeding | 11 |

Abbreviations: CT, computed tomography; ICU, intensive care unit; PCR, polymerase chain reaction.

**Supplementary Table ST2.** Patients’ distribution according to the enrollment center.

|  | | | **Intervention arm**  **(n=56)** | **Control arm**  **(n=28)*** |
| --- | --- | --- | --- | --- |
| Enrolled | Center (Country) | |  |  |
|  | 1 (Mexico) | n (%) | 4 (7.1) | 2 (7.1) |
|  | 2 (Switzerland, St. Gallen) |  | 7 (12.5) | 3 (10.7) |
|  | 3 (Brazil) |  | 7 (12.5) | 4 (14.3) |
|  | 4 (Switzerland, Zürich) |  | 10 (17.9) | 5 (17.9) |
|  | 5 (Switzerland, Basel) |  | 28 (50.0) | 14 (50.0) |
|  | Total |  | 56 (100.0) | 28 (100.0) |

* One study participant in the control arm withdrew consent immediately after randomization; therefore, the analyzed number of participants in the control arm is 27.

## Supplementary Figure S2. Course of additional laboratory parameters from baseline until day 7 according to the treatment group. Course of (A) creatinine (μmol/L, medians and interquartile range), (B) ferritin (μg/L, medians and interquartile range) and (C) D-dimer (μg/mL, median and interquartile range).


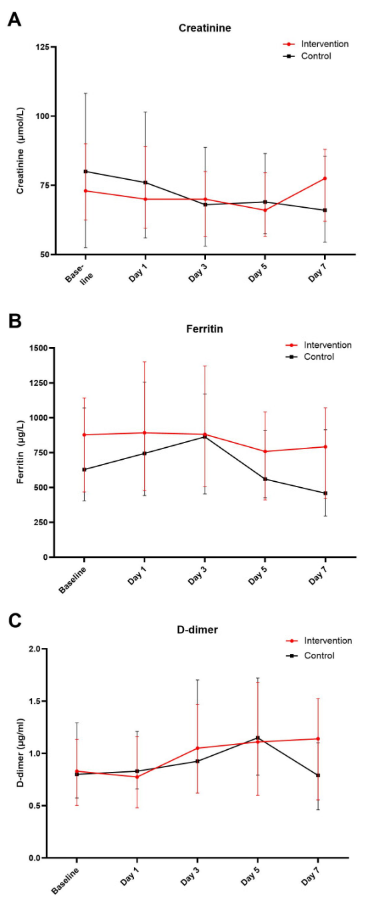


## Supplementary Figure S3. Course of complement and endothelial activation parameters from baseline until day 7 according to the treatment group. (A) Absolute change in C4 (g/L, medians and interquartile range), (B) relative change from baseline in sC5b-9 (%, medians and interquartile range; dashed line represents baseline), (C) E-selectin (ng/mL, medians and interquartile range) and (D) VCAM-1 (ng/ml, medians and interquartile range).


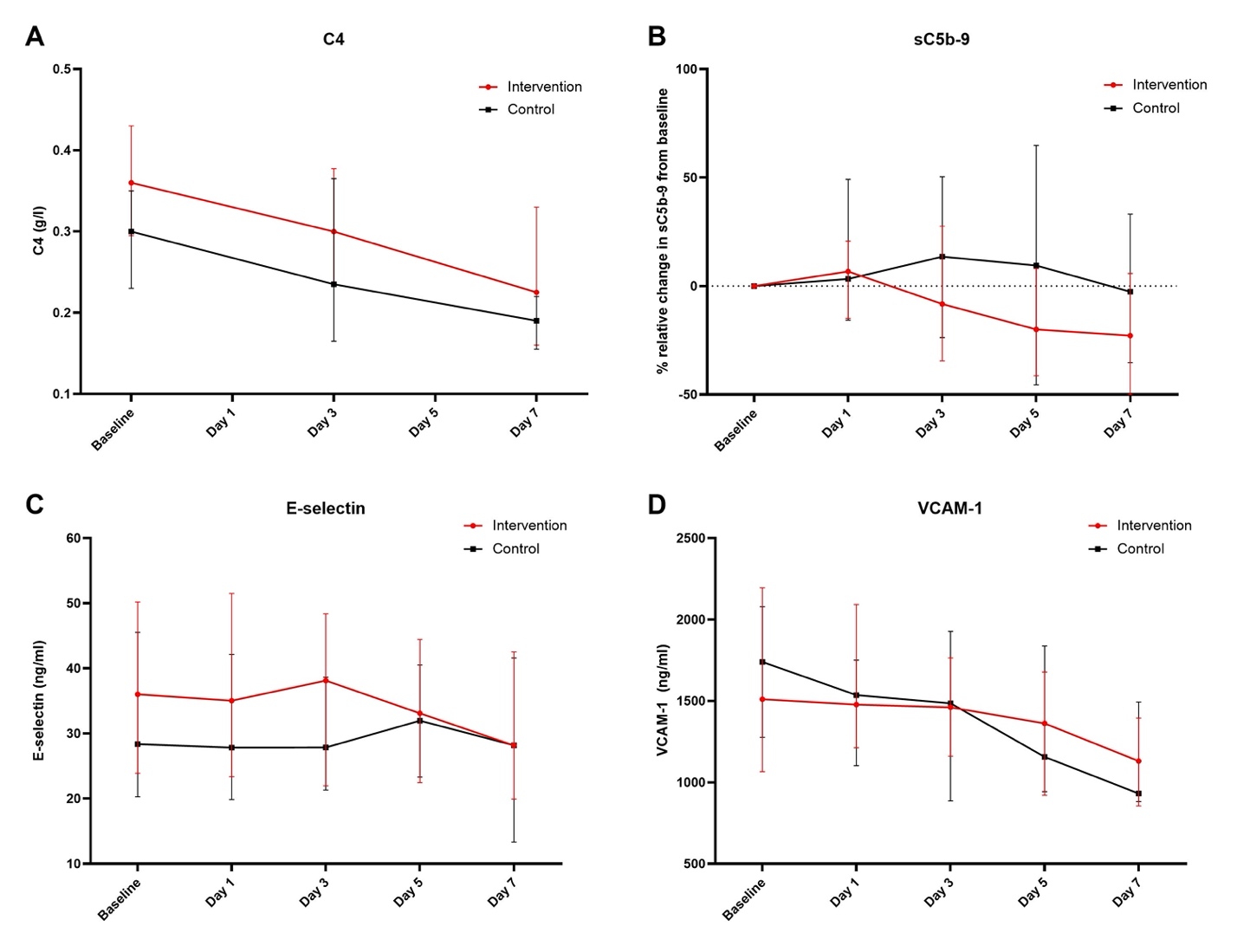


**Supplementary Table ST3.** Adverse events classified by System Organ Classification.^1^

| System Organ Class | Intervention arm (n=56) | Control arm (n=27) |
| --- | --- | --- |
| Investigations | 8 (14.3%) | 2 (7.4%) |
| Vascular disorders | 4 (7.1%) | - |
| Infections and infestations | 3 (5.4%) | - |
| Metabolism and nutrition disorders | 3 (5.4%) | - |
| Psychiatric disorders | 3 (5.4%) | - |
| Renal and urinary disorders | 2 (3.6%) | 1 (3.7%) |
| Nervous system disorders | 2 (3.6%) | - |
| Respiratory, thoracic and mediastinal disorders | 2 (3.6%) | - |
| Ear and labyrinth disorders | - | 1 (3.7%) |
| Gastrointestinal disorders | 1 (1.8%) | - |
| Injury, poisoning and procedural complications | 1 (1.8%) | - |
| Musculoskeletal and connective tissue disorders | - | 1 (3.7%) |

Multiple citations per patient are possible. Investigations refers to laboratory examinations.

^1^Carragher R, Robertson C. Assessing safety at the end of clinical trials using system organ classes: A case and comparative study. Pharm Stat. 2021;20(6):1278-1287.

**Supplementary Table ST4.** Adverse events by preferred term.

| Adverse event by Preferred Term | Intervention arm  (n=56) | Control arm  (n=27) |
| --- | --- | --- |
| Hepatic enzyme elevation | 4 (7.1%) | 1 (3.7%) |
| Hypoalbuminemia | 3 (5.4%) | - |
| Delirium | 3 (5.4%) | - |
| Blood creatinine elevation | 2 (3.6%) | - |
| Headache | 2 (3.6%) | - |
| Embolism | 2 (3.6%) | - |
| Vertigo | - | 1 (3.7%) |
| Ileus paralytic | 1 (1.8%) | - |
| Cystitis | 1 (1.8%) | - |
| Herpes simplex | 1 (1.8%) | - |
| Mucosal infection | 1 (1.8%) | - |
| Wound complication | 1 (1.8%) | - |
| International normalized ratio elevation | - | 1 (3.7%) |
| Lymphocyte count decrease | 1 (1.8%) | - |
| Neutrophil count decrease | 1 (1.8%) | - |
| Arthralgia | - | 1 (3.7%) |
| Peripheral motor neuropathy | 1 (1.8%) | - |
| Dysuria | 1 (1.8%) | - |
| Renal Failure | - | 1 (3.7%) |
| Renal injury | 1 (1.8%) | - |
| Hypoxia | 1 (1.8%) | - |
| Pulmonary cavitation | 1 (1.8%) | - |
| Hypotension | 1 (1.8%) | - |
| Phlebitis | 1 (1.8%) | - |
| Total | 30 | 5 |

Multiple citations per patient are possible.

**Supplementary Table ST5.** Serious adverse events classified by System Organ Classification and preferred term.^1^

| System Organ Class | Preferred term | Intervention arm (n=56) | Control arm (n=27) | |
| --- | --- | --- | --- | --- |
| Cardiac disorders | Total | 3 (5.4%) | - |  |
|  | Atrial fibrillation | 1 (1.8%) | - |  |
|  | Cardiac arrest | 1 (1.8%) | - |  |
|  | Cardiopulmonary failure | 1 (1.8%) | - |  |
|  | Tachycardia | 1 (1.8%) | - |  |
| Infections and infestations | Total | 6 (10.7%) | 1 (3.7%) |  |
|  | Device related infection | 2 (3.6%) | - |  |
|  | Lung infection | 1 (1.8%) | - |  |
|  | Pneumonia | 3 (5.4%) | 1 (3.7%) |  |
|  | Pneumonia bacterial | 1 (1.8%) | - |  |
|  | Sepsis | 1 (1.8%) | - |  |
|  | Septic shock | 1 (1.8%) | 1 (3.7%) |  |
| Investigations | Total | - | 1 (3.7%) |  |
|  | Blood lactic acid increased | - | 1 (3.7%) |  |
| Musculoskeletal and connective tissue disorders | Total | 1 (1.8%) | - |  |
|  | Back pain | 1 (1.8%) | - |  |
| Respiratory, thoracic and mediastinal disorders | Total | 10 (17.9%) | 2 (7.4%) |  |
|  | ARDS | 3 (5.4%) | - |  |
|  | Acute respiratory failure | 1 (1.8%) | 1 (3.7%) |  |
|  | Dyspnea | 5 (8.9%) | 1 (3.7%) |  |
|  | Pneumonitis | 1 (1.8%) | - |  |
|  | Pneumothorax | 1 (1.8%) | - |  |
|  | Pulmonary embolism | 1 (1.8%) | - |  |
|  | Respiratory failure | 2 (3.6%) | - |  |
| Surgical and medical procedures | Total | 1 (1.8%) | - |  |
|  | Oxygen therapy | 1 (1.8%) | - |  |
| Vascular disorders | Total | 2 (3.6%) | - |  |
|  | Embolism | 1 (1.8%) | - |  |
|  | Hypotension | 1 (1.8%) | - |  |

ARDS, Acute respiratory distress syndrome. Multiple citations are possible.

^1^Carragher R, Robertson C. Assessing safety at the end of clinical trials using system organ classes: A case and comparative study. Pharm Stat. 2021;20(6):1278-1287.
